# Associations of inferior frontal sulcal hyperintensities on brain MRI with cerebral small vessel disease, cognitive function, and depression symptoms

**DOI:** 10.1038/s41598-025-87493-8

**Published:** 2025-01-23

**Authors:** Marc Dörner, Malte Pfister, Anthony Tyndall, Roland von Känel, Katja Neumann, Frank Schreiber, Philipp Arndt, Erelle Fuchs, Cornelia Garz, Wenzel Glanz, Michaela Butryn, Anna-Charlotte John, Annkatrin Hildebrand, Sebastian Euler, Andreas B. Hofmann, Lena Machetanz, Johannes Kirchebner, Pawel Tacik, Alexander Grimm, Robin Jansen, Marc Pawlitzki, Solveig Henneicke, Valentina Perosa, Bendix Labeit, Emrah Düzel, Sven G. Meuth, Stefan Vielhaber, Hendrik Mattern, Jose Bernal, Stefanie Schreiber

**Affiliations:** 1 German Center for Neurodegenerative Diseases (DZNE) within the Helmholtz Association, 39120 Magdeburg, Germany; 2 Department of Consultation-Liaison-Psychiatry and Psychosomatic Medicine, University Hospital Zurich, University of Zurich, Culmannstrasse 8, Zurich, 8091 Switzerland; 3 Department of Neurology, Otto-von-Guericke University, 39120 Magdeburg, Germany; 4 Department of Neuroradiology, Clinical Neuroscience Center, University Hospital Zurich, University of Zurich, Zurich, 8091 Switzerland; 5 Department of Neuroradiology, Otto-von-Guericke University, 39120 Magdeburg, Germany; 6 Institute of Cognitive Neurology and Dementia Research, Otto-von-Guericke University, 39120 Magdeburg, Germany; 7 Department of Psychiatry, Psychotherapy, and Psychosomatics, University Hospital of Psychiatry Zurich, University of Zurich, Zurich, 8032 Switzerland; 8 Department of Forensic Psychiatry, University Hospital of Psychiatry Zurich, University of Zurich, Zurich, 8032 Switzerland; 9 Department of Parkinson’s Disease, Sleep and Movement Disorders, University Hospital Bonn, 53127 Bonn, Germany; 10 German Center for Neurodegenerative Diseases (DZNE) within the Helmholtz Association, 53127 Bonn, Germany; 11 Center for Neurology, Tuebingen University Hospital and Hertie-Institute for Clinical Brain Research, Eberhard Karls University, 72076 Tuebingen, Tuebingen, Germany; 12 Department of Neurology, Medical Faculty, Heinrich Heine University, 40225 Düsseldorf, Germany; 13 Massachusetts General Hospital, J. Philip Kistler Stroke Research Center, 02114 Boston, MA Germany; 14 Center for Behavioural Brain Sciences (CBBS), 39120 Magdeburg, Germany; 15 Biomedical Magnetic Resonance, Otto-von-Guericke University, 39120 Magdeburg, Germany; 16 Centre for Clinical Brain Sciences, The University of Edinburgh, 49 Little France Crescent, Edinburgh, EH16 4SB UK; 17 UK Dementia Research Institute Centre, University of Edinburgh, Edinburgh Bioquarter, 49 Little France Crescent, EH16 4SB Edinburgh, United Kingdom

**Keywords:** Cerebral small vessel disease, Cerebral amyloid angiopathy, Hypertensive arteriopathy, Inferior frontal sulcal hyperintensity, Glymphatic system, Fluid-attenuated inversion recovery, Magnetic resonance imaging, Alzheimer's disease, Neurodegeneration, Alzheimer's disease

## Abstract

Inferior frontal sulcal hyperintensities (IFSH) observed on fluid-attenuated inversion recovery (FLAIR) MRI have been proposed as indicators of elevated cerebrospinal fluid waste accumulation in cerebral small vessel disease (CSVD). However, to validate IFSH as a reliable imaging biomarker, further replication studies are required. The objective of this study was to investigate associations between IFSH and CSVD, and their potential repercussions, i.e., cognitive impairment and depression. We prospectively recruited 47 patients with CSVD and 29 cognitively normal controls (NC). IFSH were rated visually based on FLAIR MRI. Using different regression models, we explored the relationship between IFSH, group status (CSVD vs. NC), CSVD severity assessed with MRI, cognitive function, and symptoms of depression. Patients with CSVD were more likely to have higher IFSH scores compared to NC (OR 5.64, 95% CI 1.91–16.60), and greater CSVD severity on MRI predicted more severe IFSH (OR 1.47, 95% CI 1.14–1.88). Higher IFSH scores were associated with lower cognitive function (-0.96, 95% CI -1.81 to -0.10), and higher levels of depression (0.33, 95% CI 0.01–0.65). CSVD and IFSH may be tightly linked to each other, and the accumulation of waste products, indicated by IFSH, could have detrimental effects on cognitive function and symptoms of depression.

**Supplementary Information:**

The online version contains supplementary material available at 10.1038/s41598-025-87493-8.

## Introduction

Typically, the cerebrospinal fluid (CSF) displays a hypointense signal on T_2_-weighted fluid-attenuated inversion recovery (FLAIR) imaging. However, the inferior frontal sulci may sometimes exhibit elevated signal intensities^1^. These are referred to as inferior frontal sulcal hyperintensities (IFSH) and have been proposed to be a novel imaging biomarker indicative of impaired CSF waste clearance and glymphatic dysfunction in the brain^2–5^. This hypothesis arises because the CSF has been suggested to flow through the inferior frontal sulci and enter the meningeal lymphatics close to the cribriform plate. Consequently, the accumulation of protein, blood, and cell debris in this area could cause such hyperintensities^1^.

To date, four studies have examined IFSH as potential bioimaging marker of impaired CSF clearance in humans. Some of these studies suggested that IFSH may be associated with increasing age, as glymphatic function typically decreases with age^2,4,5^. Glymphatic dysfunction is not only an age-related phenomenon, but is also thought to play an important role in neurodegenerative diseases, such as Alzheimer’s disease (AD). One study, including cognitively normal controls (NC) and patients with mild cognitive impairment, evaluated whether IFSH might reflect amyloid or tau accumulation in the brain, but found no evidence linking IFSH to AD pathology^3^. In contrast, a large multicentre study, involving NC participants and patients on the AD continuum, established a relationship between abnormal amyloid β (Aβ) accumulation and IFSH^4^. Additionally, some of these studies found a significant association of cerebral small vessel disease (CSVD) pathology on cranial magnetic resonance imaging (cMRI), such as enlarged perivascular spaces (PVS) and periventricular and deep white matter hyperintensities (WMH), with IFSH^2,3,5^. Prior findings demonstrated that enlarged PVS are a presumed marker of impaired brain fluid and waste clearance and microvascular dysfunction. Specifically, the uptake of CSF to flush interstitial fluid and clear metabolic waste is a key function of PVS^6^. Moreover, since some WMH seem to form around PVS, impaired glymphatic function may contribute to WMH^1^. Thus, a potential link between CSVD and impaired fluid and waste clearance might result in worsening brain health, cognitive deficits or neuropsychiatric symptoms.

Although some of these studies indicated a connection between CSVD pathology and IFSH, none included a larger sample of patients diagnosed with CSVD, such as cerebral amyloid angiopathy (CAA) or hypertensive arteriopathy (HA), which belong to the group of CSVD^7^. Thus, assuming that IFSH are indicators of glymphatic dysfunction, this study has four aims. First, to compare the CSVD-related IFSH profile to that of age- and sex-matched NC. Second, to analyse the associations between IFSH and CSVD markers on cMRI. Third, to investigate possible cognitive or neuropsychiatric repercussions (symptoms of depression) of IFSH that may be attributed to the stagnation of waste proteins, blood, cellular debris and associated pathophysiological sequelae. Finally, we aimed to assess indirect mediating effects of CSVD severity on IFSH and cognition or neuropsychiatric symptoms.

## Methods

### Study design and sample

In this cross-sectional study, we prospectively enrolled patients with CAA and HA who were diagnosed and treated at the Department of Neurology at the Otto-von-Guericke University of Magdeburg between 2016 and 2022. To be included in the study, CAA patients had to meet the modified Boston criteria 1.5 for probable CAA^8^. HA diagnosis was based on the existence of deep or mixed (deep and lobar) haemorrhages^9^. NC were prospectively recruited from an existing pool of cognitively normal healthy elderly from the German Center for Neurodegenerative Diseases (DZNE). Haemorrhagic cMRI markers, such as cerebral microbleeds (CMB), cortical superficial siderosis (CSS), and intracerebral haemorrhage (ICH), were an exclusion criterion for NC. Further exclusion criteria for CAA, HA and NC participants were other central nervous system diseases, contraindications for cMRI, current or past alcohol or drug abuse, or insufficient knowledge of German language skills. All participants provided written informed consent according to the Declaration of Helsinki. The local Ethics Committee of the Otto-von-Guericke University of Magdeburg, Faculty of Medicine, approved the study (28/16).

### cMRI acquisition and CSVD markers

Neuroimaging markers were quantified according to the Standards for Reporting Vascular Changes on Neuroimaging 2 (STRIVE-2) criteria through standardised 3 Tesla (T) cMRI, and assessments were blinded to all demographic and clinical data^10^. These included haemorrhagic (CMB, CSS, ICH) and non-haemorrhagic markers (WMH, PVS, lacunes) as well as global cortical atrophy (GCA) and medial temporal atrophy (MTA). All markers were assessed by a trained investigator (M.P.) according to previously established scales^11–16^. To determine intra- and interrater reliability, in 10 randomly chosen MRI scans CSVD markers were scored by two independent raters (M.P., A.T.) twice within a period of several weeks, blinded to all clinical data and demographics.

The ordinal summary CSVD score ranges between 0 and 6 points, whereas one point is given for the presence of lacunes, 1–4 CMB, severe basal ganglia PVS (> 20), and moderate WMH (sum of deep and periventricular WMH grade 3–4) each^11^. Two points are given for the presence of > 5 CMB or severe WMH (sum of deep and periventricular WMH grade 5–6)^11^. HA severity is scored from 0 to 4 points: one point is allocated for the presence of lacunes, deep WMH grade 2–3 or periventricular WMH grade 3, the presence of at least one deep CMB, and moderate to severe basal ganglia PVS (> 10)^11^. To evaluate the overall CAA burden, we applied the total CAA severity score (range 0 to 6 points) through the following scale^17^: one point is given for the presence of (a) 2–4 lobar CMB, (b) high degree centrum semiovale (CSO) PVS (> 20 CSO PVS), (c) deep WMH grade ≥ 2 or periventricular WMH = 3 or (d) focal CSS, respectively. Two points are given for ≥ 5 lobar CMB and multifocal CSS each.

Measurements were performed using a Siemens Verio MRI system (Siemens, Erlangen, Germany) with a Siemens 32-channel array coil. The protocol included a T_2_-weighted 3D FLAIR sequence (voxel size 1 × 1 × 1 mm^3^, echo time (TE) 395 ms, repetition time (TR) 5000 ms, inversion time (TI) 1800 ms, flip angle (FA) 120°, T_2_-selective inversion recovery, receiver bandwidth (RBW) 781 Hz/px; generalised autocalibrating partially parallel acquisition (GRAPPA) factor 2, 24 reference lines), to localise lacunes and evaluate WMH, MTA and IFSH. A T_2_-weighted 2D turbo spin-echo sequence permitted us to identify PVS and GCA (voxel size 0.5 × 0.5 × 2 mm^3^, TE 63 ms, TR 6500 ms, FA 120°, RBW 222 Hz/px; GRAPPA factor 2, 24 reference lines). CMB, ICH and CSS were rated using a susceptibility-weighted 3D gradient-echo pulse sequence (voxel size 1 × 1 × 2 mm^3^, TE 20 ms, TR 28 ms, FA 17°, RBW 100 Hz/px; GRAPPA factor 2, 24 reference lines). The use of imaging acceleration (such as GRAPPA for FLAIR) in the study design was motivated to enable patient compliance throughout scanning. FLAIR sequences lasted approximately seven minutes. Scanning time totalled ∼45 min.

### Measurements

We assessed demographics (age, sex), years of education, clinical diagnoses, and arterial hypertension (i.e., former diagnosis based on medical records and/or use of antihypertensive medication for blood pressure control^18^). Additionally, cognitive function was measured by the Mini-Mental State Examination (MMSE) total score^19^, and the Geriatric Depression Scale-Short Form (GDS-SF) was used to evaluate symptoms of depression. The optimal cut-offs of the GDS-SF are ≥ 5 (minor depressive disorder) and ≥ 10 points (major depressive disorder)^20^. The biomarker-based Aβ, tau, and neurodegeneration (ATN) framework was applied to assess concomitant AD pathology derived from CSF data according to the National Institute on Aging - Alzheimer’s Association^21^. Aβ_42/40_ ratio was considered for the determination of Aβ positivity (A+), phosphorylated tau (p-tau) for tau positivity (T+), and total tau (t-tau) or neurofilament light chain (NF-L) for neurodegeneration (N+; **Supplement 1**). A + T + N + or A + T + N- were considered as AD, A + T-N- as Alzheimer’s pathologic change, and A + T-N + as Alzheimer’s and concomitant suspected non Alzheimer’s pathologic change, placing these individuals on the AD continuum^21^.

### Method of IFSH rating

**Fig. 1:**
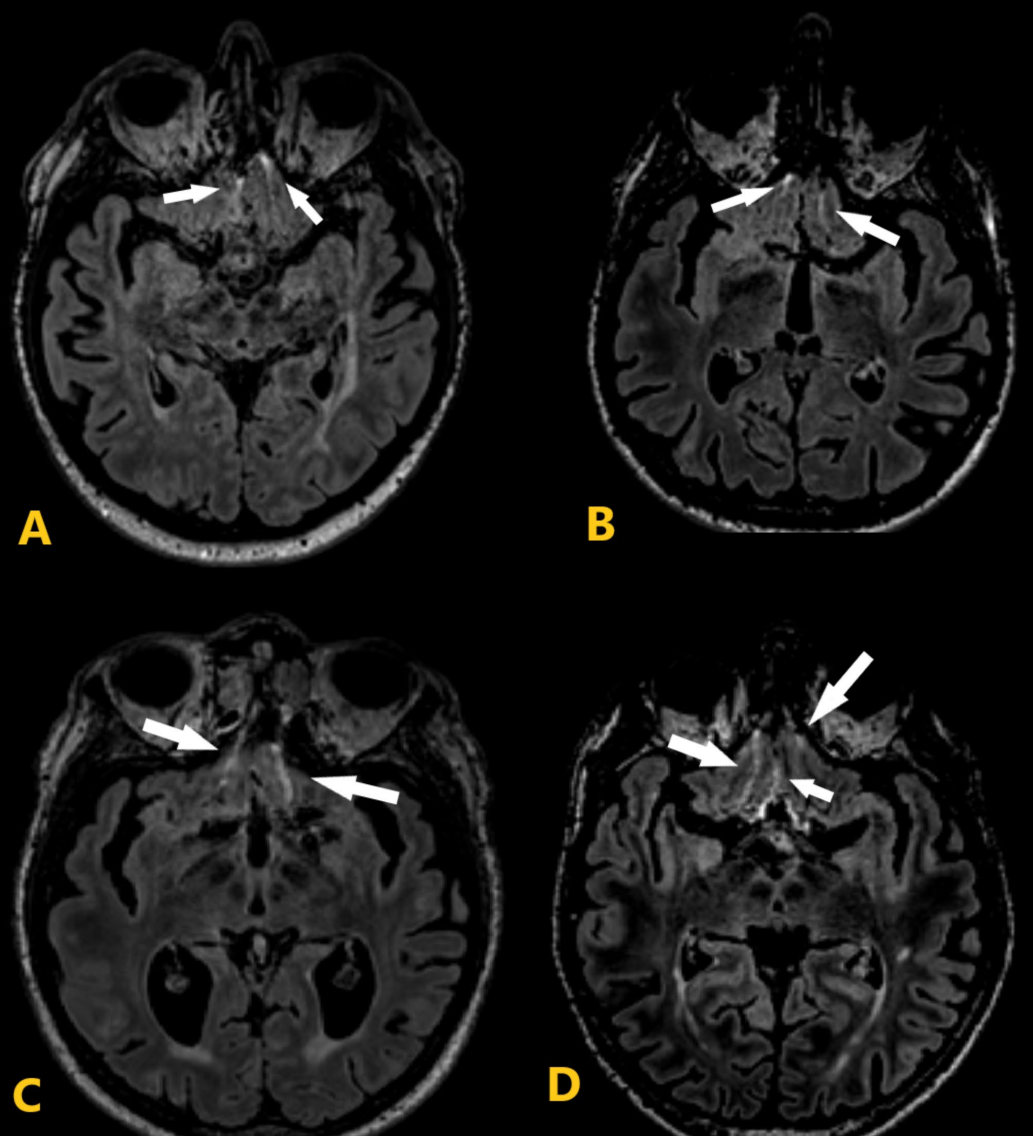
Example of Inferior Frontal Sulcal Hyperintensity (IFSH) on fluid-attenuated inversion recovery (FLAIR) images. The IFSH score in each of the three inferior frontal sulci was assessed above the reference slice. (**A**) The IFSH total score was 2 (see white arrows: one point for the central sulcus and one point for the left sulcus). (**B**) The IFSH total score was 3 (1 point for the right sulcus and two points for the left sulcus). (**C**) The IFSH total score was 5 (two points for the right sulcus and three points for the left sulcus). (**D**) The total IFSH score was 6 (three points for the right, two points for the central sulcus, and one point for the left sulcus.

IFSH were rated according to the user guide by Lim et al.^22^. They were defined as hyperintense CSF signals on FLAIR in one or more of the three inferior frontal sulci, which encompass the central sulcus between the gyri recti and both the right and left olfactory sulci. In a first step, utilising multi-planar reconstruction (MPR), all FLAIR sequences were orientated parallel to the floor of the anterior cranial fossa, thereby displaying the sulci on one plane. Then, the reference slice clearly displaying all three sulci was identified. Each sulcus above the reference slice was evaluated over multiple axial slices, and the maximum sulcal length affected was documented. Scores for each sulcus range between 0 and 3 (0 = none of the sulcus affected; 1 = less than half of the sulcus length affected; 2 = at least half of the sulcus length affected; 3 = most or whole of the sulcus length affected). The total IFSH score was calculated by adding all three sulci scores (range 0 to 9 points). Figure 1 illustrates examples of different IFSH scores. To avoid estimation and small cell problems due to sparse data, we divided the total IFSH score into three categories (0–2, 3–4, 5–9 points) according to the distribution of the total scores among our participants. This approach is in line with other studies^2–4^.

A trained rater and resident in Neurology (M.D.), using Mango software^23^, assessed the IFSH. The same rater as well as another Neurology resident (M.P.) examined MRI scans of *n* = 37 randomly chosen participants a second time several weeks after the initial MRI analyses. Both were blinded to all clinical and demographical data. Cohen’s kappa demonstrated good to excellent intra- and inter-rater consistency for the right sulci (kappa_intra_ = 0.67, kappa_inter_ = 0.73), central sulci (kappa_intra_ = 0.81, kappa_inter_ = 0.86), and left sulci (kappa_intra_ = 0.80, kappa_inter_ = 0.65).

### Statistical analysis

We used IBM SPSS Statistics for Windows, Version 29 (Armonk, NY: IBM Corp), for statistical analysis. Mean and median scores, standard deviation, and relative and absolute distributions were used to describe patient characteristics. Non-normally distributed continuous variables were compared applying a Mann-Whitney U or Kruskal-Wallis test, and a chi-square or Fisher’s exact test, where appropriate, was performed to evaluate differences between non-normally distributed categorical variables.

Regression analyses addressed two main aspects: the first aimed to identify factors that contributed to or correlated with IFSH (IFSH as the outcome variable), and the second focused on exploring the relationship between IFSH and its potential effects, specifically cognition or symptoms of depression (IFSH as predictor). First, a multivariate ordinal logistic regression analysis was conducted, since the IFSH score as dependent variable was ordered in three categories (0–2, 3–4, 5–9; see 2.4), and group status (CAA/HA vs. NC) was used as independent variable. In a second step, another multivariate ordinal logistic regression analysis tested the effect of CSVD MRI markers and brain atrophy (GCA, MTA) on the IFSH categorical score as dependent variable. To assess cognitive or neuropsychiatric (symptoms of depression) repercussions of IFSH, we applied a multivariate linear regression with the IFSH categorical score as independent variable, and the total MMSE and GDS-SF continuous scores as dependent variables. Lastly, we utilised the PROCESS regression path analysis modeling tool for SPSS to estimate mediation effects of CSVD disease severity assessed with MRI on IFSH and cognitive function or symptoms of depression. All regression and mediation models included both CAA and HA patients as well as NC participants, divided the IFSH score in categories (see 2.4), and were adjusted for age, sex, years of education, and arterial hypertension (yes or no), unless otherwise noted. Collinearity statistics did not identify any issues of multicollinearity. Significance level was set at *p* < 0.05 (two-sided p-value). To correct p-values for multiple testing, we used the method described by Benjamini and Hochberg^24^.

## Results

### Study sample

In this study, we included 76 age- and sex-matched participants, i.e., 21 CAA patients, 26 HA patients, and 29 NC (see Table 1). CAA and HA participants had significantly more often a history of arterial hypertension, lower cognitive status and higher depression levels than NC. Furthermore, IFSH scores for the right, left, central sulcus, and the total IFSH sum scores were significantly lower in NC compared to HA and CAA. A Mann-Whitney U, chi-square and Fisher’s exact test did not indicate any statistically significant differences between CAA and HA subgroups. The leading clinical diagnosis in HA and CAA patients was transient ischemic attack/ stroke (51.1%), followed by cognitive decline and ICH (both 21.3%). Only three patients (6.3%) suffered from transient focal neurological episodes.

**Table 1:** Sociodemographic and clinical characteristics of the study sample. Information regarding history of arterial hypertension was available for most but not all participants (75/76).

	Overall (*n* = 76)	CAA (*n* = 21)	HA (*n* = 26)	NC (*n* = 29)	p-value (*p* < 0.05)	*p*-value^c^ (*p* < 0.05)
Age, y	69.89 (8.4)	69.57 (11.5)	69.92 (7.4)	70.10 (6.7)	0.920^**a**^	0.920
Male, n (%)	45 (59.2)	13 (61.9)	17 (65.4)	15 (51.7)	0.584^b^	0.713
Years of education	14.78 (2.8)	14.22 (2.58)	14.10 (3.2)	15.75 (2.5)	**0.047** ^**a**^	0.064
Arterial hypertension, n (%)	59 (77.6)	17 (85.0)	25 (96.2)	17 (58.6)	**0.002** ^**b**^	**0.004**
MMSE total score	26.99 (2.8)	25.94 (3.3)	25.88 (3.0)	28.67 (1.1)	**< 0.001** ^**a**^	**0.011**
GDS-SF total score	2.64 (2.6)	3.94 (2.1)	3.81 (3.0)	0.85 (0.9)	**< 0.001** ^**a**^	**0.011**
*Clinical diagnoses*,* n (%)* ICH	*n* = 47 10 (21.3)	6 (28.6)	4 (15.4)		0.803^b^	0.883
TIA/Stroke	24 (51.1)	10 (47.6)	14 (53.8)			
TFNE	3 (6.3)	1 (4.8)	2 (7.7)			
Cognitive decline	10 (21.3)	4 (19.0)	6 (23.1)			
*IFSH score* Right sulcus	1.18 (0.7)	1.33 (0.7)	1.46 (0.8)	0.83 (0.5)	**0.005** ^a^	**0.009**
Central sulcus	0.91 (0.7)	1.10 (0.7)	1.19 (0.8)	0.52 (0.5)	**0.002** ^a^	**0.004**
Left sulcus	1.34 (0.6)	1.38 (0.6)	1.58 (0.7)	1.10 (0.5)	**0.027** ^**a**^	**0.042**
Total IFSH sum score	3.42 (1.7)	3.76 (1.4)	4.23 (2.0)	2.45 (1.1)	**< 0.001** ^**a**^	**0.011**

Note: n: number. y: years. CAA: cerebral amyloid angiopathy. HA: hypertensive arteriopathy. NC: cognitively normal control. MMSE: Mini-Mental State Examination. GDS-SF: Geriatric Depression Scale-Short Form. ICH: intracerebral haemorrhage. TIA: transient ischemic attack. TFNE: transient focal neurological episode. IFSH: Inferior Frontal Sulcal Hyperintensity. Values are mean (standard deviation) unless otherwise noted. Significant p-values are marked bold. p-values are based on a ^a^Kruskal-Wallis test for continuous variables, and a ^b^chi-square or Fisher’s exact test for categorical variables. ^c^ Corrected p-values using Benjamini’s and Hochberg’s method.

CSF data were available in 29 patients (14 CAA/ 15 HA, 61.7%), of whom four CAA cases demonstrated AD (one A + T + N-, three A + T + N+). Three patients were A-T-N- (one CAA, two HA), six A + T-N- (AD pathologic change; three CAA, three HA), ten A-T-N+ (two CAA, eight HA), four A-T + N+ (three CAA, one HA), and two A + T-N+ (Alzheimer’s and concomitant suspected non Alzheimer’s pathologic change; one CAA and one HA). The sum scores of the right (*p* = 0.464), left (*p* = 0.570), and central sulcus (*p* = 0.170), and the combined total IFSH sum score (*p* = 0.699) did not indicate any significant differences between biomarker profiles.

Intra- and interrater reliability (kappa_intra_ = 0.86; kappa_inter_ = 0.92) of MRI CSVD marker assessments were excellent.

### Associations between IFSH, group status and MRI CSVD markers

The association between the IFSH categorical score as dependent variable and group status (CAA/HA vs. NC) as independent variable is illustrated in Table 2. A multivariate ordinal logistic regression analysis suggested that CAA or HA diagnosis predicted significantly higher IFSH scores, even after adjusting for several covariates. The association of group status remained significant for CAA after excluding HA patients (odds ratio (OR) 7.72, 95% confidence interval (CI) 1.99 to 29.99, *p* = 0.003), and, vice versa, for HA patients after the exclusion of CAA patients (OR 5.56, 95% CI 1.57 to 19.49, *p* = 0.008). Another regression analysis, replacing the group status (CAA/HA vs. NC) with the clinical diagnoses of CAA and HA patients (i.e., ICH, transient ischemic attack/ stroke, transient focal neurological episode, or cognitive decline) as independent variable, did not indicate a significant association with IFSH (results not shown).

**Table 2:** Associations between IFSH categorical score and group status.

	Multivariate		
Variables	OR (95% CI)	*p*-value (*p* < 0.05)	*p*-value^a^ (*p* < 0.05)
Age	1.00 (0.61 to 1.06)	0.831	0.831
Female sex	0.87 (0.34 to 2.21)	0.782	0.977
Years of education	1.08 (0.91 to 1.28)	0.365	0.608
Arterial hypertension	2.23 (0.66 to 7.53)	0.194	0.485
CAA/HA vs. NC	5.64 (1.91 to 16.60)	**0.002**	**0.01**

Note: OR: odds ratio. CI: confidence interval. Significant p-values are marked bold. ^a^ Corrected p-values using Benjamini’s and Hochberg’s method.

In a second step, we separately analysed the ordinal summary CSVD score, the total HA, the total CAA, and the MTA and GCA scores as independent variables and the IFSH categorical score as dependent variable. Of note, the odds of having higher IFSH categorical scores were higher in subjects with greater CSVD (OR 1.47, 95% CI 1.14–1.88, *p* = 0.002), HA (OR 1.57, 95% CI 1.06–2.32, *p* = 0.023), and CAA severity (OR 1.77, 95% CI 1.29–2.44, *p* < 0.001). However, brain atrophy (MTA: OR 0.90, 95% CI 0.60–1.37, *p* = 0.650; GCA: OR 0.85, 95% CI 0.49–1.49, *p* = 0.589) did not predict IFSH on brain MRI.

Third, we explored what might be the driving markers related to higher IFSH categorical scores within CSVD, HA, and CAA pathology, i.e., haemorrhagic (CMB, CSS, ICH; Step 1) as well as non-haemorrhagic MRI markers (enlarged PVS in the centrum semiovale and basal ganglia, WMH, lacunes; Step 2; Table 3). Interestingly, a higher number of ICH was linked to significantly higher IFSH categorical scores. A history of arterial hypertension was also related to more severe IFSH, while the other variables were not associated with higher IFSH categorical scores.

**Table 3:** Associations between IFSH categorical score and CSVD markers.

	Step 1 Multivariate		Step 2 Multivariate	
Variables	OR (95% CI)	*p*-value	OR (95% CI)	*p*-value
Age	1.00 (0.95 to 1.06)	0.791	1.00 (0.95 to 1.06)	0.854
Female sex	0.87 (0.34 to 2.22)	0.774	1.02 (0.40 to 2.62)	0.952
Years of education	1.03 (0.87 to 1.22)	0.698	1.07 (0.90 to 1.29)	0.401
Arterial hypertension	4.71 (1.30 to 16.99)	**0.018**	3.59 (1.04 to 12.55)	**0.043**
CMB total count	1.00 (0.99 to 1.01)	0.510		
CSS sum score (0–4)	0.94 (0.69 to 1.26)	0.697		
ICH total count	2.91 (1.15 to 7.46)	**0.024**		
PVS CSO (0–4)			1.28 (0.74 to 2.22)	0.372
PVS BG (0–4)			1.44 (0.70 to 2.94)	0.312
WMH sum score (0–6)			0.93 (0.63 to 1.37)	0.723
Lacunes total count			1.08 (0.95 to 1.23)	0.221

Note: OR: odds ratio. CI: confidence interval. CSVD: cerebral small vessel disease. CMB: cerebral microbleeds. CSS: cortical superficial siderosis. PVS: enlarged perivascular spaces. CSO: centrum semiovale. BG: basal ganglia. WMH: white matter hyperintensities. Significant p-values are marked bold.

### Associations between IFSH and cognitive function and symptoms of depression

A multivariate linear regression analysis explored a potential link between the IFSH categorical score as independent variable, and cognitive function (MMSE total score) or symptoms of depression (GDS-SF total score) as dependent variables, adjusting for age, sex, years of education, arterial hypertension, and diagnoses of CAA and HA. More severe IFSH predicted lower cognitive function (mean difference − 0.96, 95% CI -1.81 to -0.10, *p* = 0.028). NC participants showed significantly higher MMSE total scores than CAA or HA participants (mean difference 1.89, 95% CI 0.51–3.28, *p* = 0.008). The inclusion of the IFSH categorical score as independent variable led to an increased variability explained by the regression model: adjusted R^2^ 23.8% vs. 25.3% (see **Supplement 2**). Additionally, participants with higher IFSH categorical scores were more likely to show symptoms of depression (mean difference 0.33, 95% CI 0.01–0.65, *p* = 0.039).

**Fig. 2:**
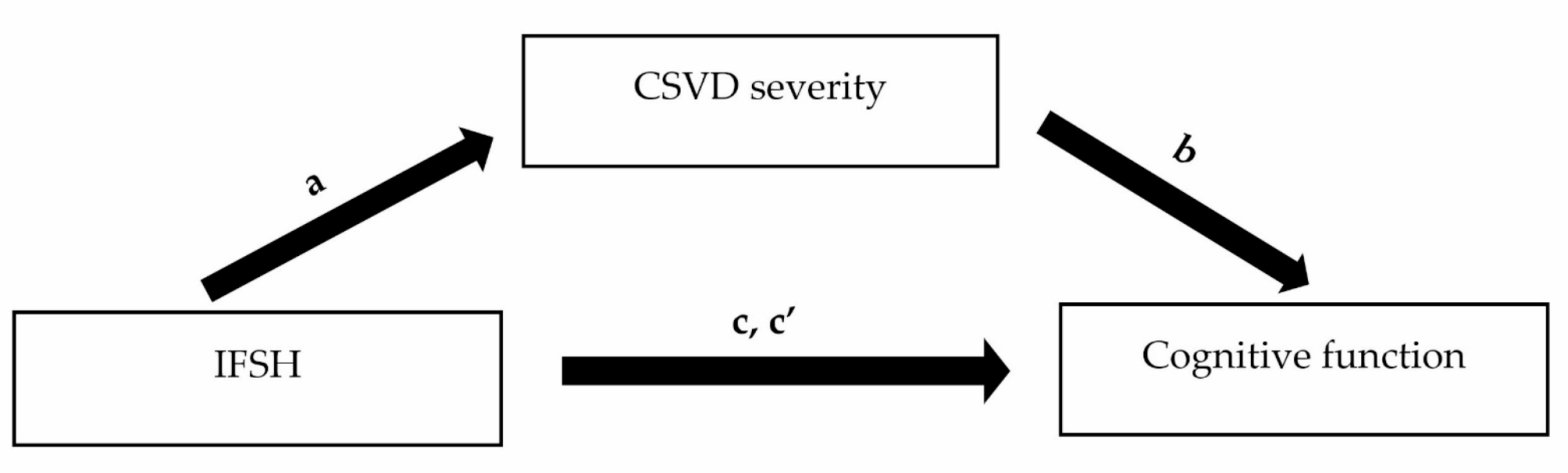
Mediation model testing indirect effects of magnetic resonance imaging cerebral small vessel disease severity (ordinal summary CSVD score) on inferior frontal sulcal hyperintensities (IFSH categorical score) and cognitive function (Mini-Mental State Examination total score). IFSH was the independent, cognitive function the dependent variable, and CSVD severity the mediator. Covariates were age, sex, years of education, and arterial hypertension. (**a**) effect of IFSH on CSVD severity (0.99, 95% confidence interval (CI) 0.38 to 1.60, p = 0.001). (**b**) effect of CSVD severity on cognitive function (-0.51, 95% CI -0.81 to -0.22, p < 0.001). (**c’**) direct effect of IFSH on cognitive function (-0.44, 95% CI -1.42 to 0.53, *p* = 0.369). (**c**) total effect of IFSH on cognitive function (-0.96, 95% CI -1.87 to -0.05, *p* = 0.038).

Figure 2displays a mediation model highlighting indirect effects of CSVD severity on IFSH as independent and cognitive function as dependent variable. In this model, CSVD severity fully mediated the relationship between those variables (indirect effect − 0.51, 95% CI -1.07 to -0.17). Another mediation model testing indirect effects of CSVD severity suggested a full mediation on IFSH and symptoms of depression (indirect effect 0.55, 95% CI 0.20 to 1.03). Depressive symptoms and cognitive function did not show any signs of collinearity.

## Discussion

In the present study, patients with CSVD exhibited significantly higher IFSH scores compared to NC. This relationship seemed to be driven by CSVD severity, i.e., higher CSVD, HA, and CAA scores on MRI and more frequent ICH. More severe IFSH were associated with lower cognitive function and greater symptoms of depression, even after adjusting for age, sex, years of education and arterial hypertension. Furthermore, our findings suggest that CSVD severity might mediate the relationship between IFSH and cognitive function or depressive symptoms, and that IFSH and CSVD severity could potentially have reciprocal effects.

Our observation that participants diagnosed with CAA or HA demonstrated more elevated signal intensities in the inferior frontal sulci than NC implies that IFSH on standardised 3T FLAIR MRI are not solely an artefact but rather a manifestation of decreasing brain health. This aligns with previous studies that investigated IFSH in humans^2–5^. FLAIR sulcal hyperintensities in other brain regions have so far been attributed to the accumulation of cell debris^25^, waste proteins^26,27^, or blood^28^ caused by local pathologies such as infection or stroke^29^. A disruption in the blood-brain barrier has been considered as potential mechanism for allowing substances to enter the CSF. However, the brain’s fluid and waste clearance system is not only managed by blood vessels supplying the brain. Recent research indicated that the glymphatic system, a non-blood-vessel brain circulation flushing the brain, and draining interstitial fluid and CSF as well as waste from the cranial cavity, may be another key element for maintaining neuronal and glial function^1^. Although the mechanism leading to IFSH is unknown, experiments have shown that some CSF drains through the cribriform plate to nasal lymphatics, and thus would allow waste accumulation directly located above the cribriform plate in the inferior frontal sulci^30,31^.

Indeed, prior studies suggested that CSVD pathology, such as deep or periventricular WMH^3,5^ or enlarged PVS in the centrum semiovale^2,5^, might contribute to glymphatic dysfunction and the buildup of toxic waste in the inferior frontal sulci. These findings are not surprising, since PVS are known to flush interstitial fluid and waste from the brain^1,32^. On the other hand, dilation of PVS is indicative of PVS and microvascular dysfunction, and subsequently impaired waste clearance^1^. Additionally, reduced cerebrovascular reactivity in patients with more severe WMH has been suggested to impede CSF flow in CSVD^33^, since cerebrovascular pulsation plays a pivotal role for CSF transportation in the glymphatic system^34^. However, these results are partly inconsistent with our observations. In contrast to previous studies^2,3,5^, we could not find significant associations between single markers of CSVD severity and IFSH, but rather the combination of haemorrhagic and non-haemorrhagic CSVD markers, i.e., the total CSVD, CAA, and HA scores pointed towards more intense IFSH. This observation suggests that there might be several sources involved in the development of IFSH, such as local brain damage caused by CMB or CSS, and together these pathologies could then lead to a steady accumulation of waste products. Notably, our study investigating IFSH in humans is the first that involved a larger sample of patients diagnosed with CAA or HA and that applied the ordinal summary CSVD, the total CAA and total HA scores, potentially allowing more disease-specific assumptions. Interestingly, ICH (macrobleeds) also predicted higher IFSH scores, which is in line with prior findings that identified blood as a cause of sulcal hyperintensities on FLAIR MRI^28^. This may also support the hypothesis that both blood-brain barrier disruption and glymphatic dysfunction could induce IFSH.

As opposed to prior findings^2,4,5^, arterial hypertension was linked to elevated signals in the inferior frontal sulci in our study. High blood pressure, a major risk factor for CSVD development, changes vascular stiffness and reduces the continuous forward flow of CSF particularly among the PVS, which consequently could foster debris deposition and obstruct the PVS^35^. Although brain atrophy reflects a loss of neurons and the exposition of larger molecules, cell debris or proteins, which may deposit in the inferior frontal sulci, we did not find sufficient evidence linking atrophy of the brain to higher IFSH scores. While our findings are in line with previous ones^3^, future studies are warranted to examine this relationship in more detail. AD pathology is a common finding in CSVD^7^, and a recent study indicated an association between Aβ accumulation and IFSH^4^. However, the current study did not suggest any IFSH differences between CSF biomarker profiles, i.e., between individuals on the AD continuum and those who did not present AD pathology. This observation confirms another study that could not identify a link between amyloid or tau accumulation in the brain and IFSH^3^. Certainly, more studies are needed to illuminate a possible relation.

Since IFSH have been suggested to be an imaging biomarker of impaired glymphatic function, there are likely clinical implications due to the accumulation of toxic waste products and worsening brain health^2–5^. Contrary to previous studies^4,5^, we found IFSH to be associated with lower cognitive function, and, as a novelty, to be indicative of higher levels of depression - this independently of CAA or HA diagnosis. These inconsistent findings might be explained due to different patient populations^4,5^ and the assessment of cognitive function via telephone in another study^5^. In our study, this relationship was mediated by CSVD severity on MRI scans, which is not surprising given that CSVD is associated with cognitive decline and neuropsychiatric symptoms^7,36^. While our regression models suggested that CSVD may contribute to glymphatic dysfunction and IFSH, mediation analyses also implied that waste accumulation, as potentially measured by IFSH, may reciprocally impact the development of CSVD. This could introduce a vicious cycle by increasing stenosis and occlusion of cerebral microvasculature, exacerbating ischemic, hypoxic and inflammatory reactions, and thereby contributing to CSVD, cognitive decline and neuropsychiatric symptoms^37–39^.

This study has several limitations. The cross-sectional design does not allow for causal inferences from the obtained results. Therefore, longitudinal studies are needed to determine the causal relationships between IFSH and other variables. To date, the exact source of IFSH remains elusive, because it is not possible to obtain CSF samples from the inferior frontal sulci. Moreover, the existence of imaging artefacts (e.g. imperfect CSF suppression, magnetic susceptibility or flow artefacts) cannot be completely ruled out due to the IFSH location being prone to artefacts in this area^40^. Patients’ characteristics, such as body mass index, head size, or brain atrophy, and other MR-related aspects, such as field inhomogeneities and imperfect inversion, might also cause these artefacts^41,42^. Additionally, GRAPPA could cause residual aliasing artefacts or noise amplification. While improving the inversion recovery, e.g., by increasing the radiofrequency bandwidth or using alternative inversion approaches, the here used T_2_-selective inversion could prevent imperfect inversion^42,43^, and we did not observe any artefacts in the IFSH area beside those already noted by Lim et al.^22^ (see also **Supplement 3**). Nevertheless, an imperfect T_2_-selective inversion due to field inhomogeneities cannot be ruled out completely as origin of the signal hyperintensity^43^. In addition, the study sample was relatively small, although comparable to other studies^2,3,5^. Another limitation is the restricted applicability of the MMSE and GDS-SF for the assessment of cognitive function and depression, which are rather screening instruments and thus cannot replace a diagnosis based on clinical interviews or a comprehensive neuropsychological test battery.

## Conclusions

This cross-sectional study suggests a link between higher IFSH scores and patients diagnosed with CSVD, and that this relationship might be driven by CSVD severity assessed with MRI. Moreover, IFSH appear to have cognitive and neuropsychiatric sequelae, possibly due to the accumulation of waste products. CSVD pathology might mediate this relationship, highlighting the potential complex interplay between these variables. However, given that the definite nature of IFSH remains unclear, our findings should be interpreted with caution. Future histopathological studies, advanced neuroimaging techniques, and studies including diverse patient populations may provide further inside into this question.

## Electronic supplementary material

Below is the link to the electronic supplementary material.

Supplementary Material 1

## Data Availability

The data presented in this study are available on request from the corresponding author.
