# Supplementary material for: Associations of inferior frontal sulcal hyperintensities on brain MRI with cerebral small vessel disease, cognitive function, and depression symptoms
Source: Sci Rep. 2025 Jan 23;15:2999. doi: 10.1038/s41598-025-87493-8 (PMC11758024; doi:10.1038/s41598-025-87493-8)
Supplement: Supplementary file 1 — Supplementary Material 1 [file 41598_2025_87493_MOESM1_ESM.docx]

**Supplement 1. CSF examination**

No longer than 20 minutes after lumbar puncture, CSF samples were centrifuged at 4 °C, aliquoted and stored at -80 °C until analysis. Thresholds for CSF markers of neurodegeneration were set for Aβ_42/40_ ratio at ≤ 0.05 (until December 2019) or ≤ 0.069 (since January 2020), for t-tau at >350 pg/ml (until December 2019) or >404 pg/ml (since Januray 2020), for neurofilament light chain (NF-L) at >3643 pg/ml, and for p-tau at >70 pg/ml (until December 2019) or >56.5 pg/ml (since January 2020), according to inhouse standards.

**Supplement 2.** Associations between IFSH categorical score and cognitive function (MMSE total score).

|  | **Step 1**  **Multivariate** | | **Step 2**  **Multivariate** | |
| --- | --- | --- | --- | --- |
| **Variables** | **Mean difference (95% CI)** | **p-value** | **Mean difference (95% CI)** | **p-value** |
| Age | -0.01 (-0.08 to 0.05) | 0.618 | -0.01 (-0.08 to 0.05) | 0.636 |
| Male sex | -0.63 (-1.81 to 0.53) | 0.281 | -0.61 (-1.78 to 0.55) | 0.300 |
| Years of education | 0.08 (-0.13 to 0.30) | 0.428 | 0.10 (-0.11 to 0.31) | 0.362 |
| Arterial hypertension (no) | 0.40 (-1.11 to 1.93) | 0.596 | 0.28 (-1.24 to 1.82) | 0.708 |
| NC vs CAA/ HA | 2.19 (0.90 to 3.48) | **0.001** | 1.89 (0.51 to 3.28) | **0.008** |
| IFSH categorical score |  |  | -0.96 (-1.81 to -0.10) | **0.028** |
| Adjusted R^2^ | 0.238 |  | 0.253 |  |

Note: CI: confidence interval. IFSH: Inferior Frontal Sulcal Hyperintensity. MMSE: Mini-Mental State Examination. NC: cognitively normal control. CAA: cerebral amyloid angiopathy. HA: hypertensive arteriopathy. Significant p-values are marked bold.


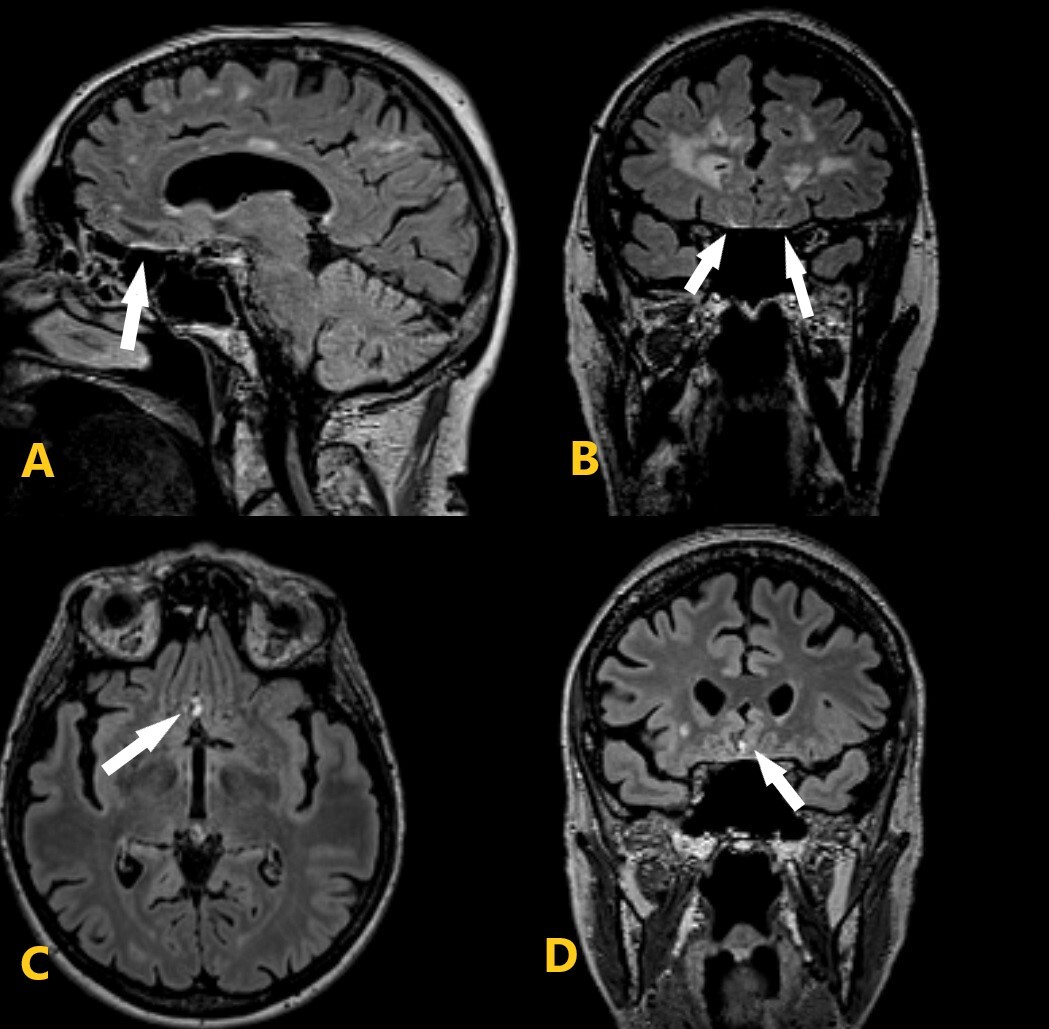


**Supplement 3.** Inferior Frontal Sulcal Hyperintensity (IFSH) on fluid-attenuated inversion recovery (FLAIR) images in different planes and artefacts in the middle sulcus. A: white arrow points towards the IFSH in the sagittal plane. B: white arrows point towards the IFSH in the coronal plane. C and D: the white arrows point towards a single focus of hyperintensity in the posterior area of the middle sulcus. These hyperintensities are associated with the optic chiasm or the presence of blood vessels and were not considered for the scoring of the IFSH. Hyperintensities in A and B were rated as IFSH due to their elongated shape closely following the sulcus’ structure. If these were artefacts, the hyperintensities in A and B would presumably have a more spherical appearance and propagate into the brain parenchyma, whis was not the case. Nevertheless, an imperfect T_2_-selective inversion due to field inhomogeneities cannot be ruled out completely as origin of the signal hyperintensity.
